# Effect of *Eurycoma longifolia* standardised aqueous root extract–Physta^®^ on testosterone levels and quality of life in ageing male subjects: a randomised, double-blind, placebo-controlled multicentre study

**DOI:** 10.29219/fnr.v65.5647

**Published:** 2021-05-19

**Authors:** Sasikala M. Chinnappan, Annie George, Pragya Pandey, Govinda Narke, Yogendra Kumar Choudhary

**Affiliations:** 1 Biotropics Malaysia Berhad, Shah Alam, Selangor, Malaysia; 2 Oriana Hospital, Ravindrapuri, Varanasi, Uttar Pradesh, India; 3 Lokmanya Multi-Specialty Hospital, Pradhikaran, Nigdi, Pune, Maharashtra, India; 4 Ethix Pharma, Mowa, Raipur, Chhattisgarh, India

**Keywords:** Eurycoma longifolia, *testosterone*, *quality of life*, *ageing*, *Physta*^®^

## Abstract

**Background:**

Low testosterone levels cause physiological changes that compromise the quality of life in ageing men. A standardised water extract from the root of *Eurycoma longifolia* (EL), known as Physta^®^, is known to increase testosterone levels.

**Objective:**

To evaluate the safety and efficacy of Physta^®^ in improving the testosterone levels and quality of life in ageing male subjects.

**Design:**

This randomised, double-blind, placebo-controlled study enrolled 105 male subjects aged 50–70 years with a testosterone level <300 ng/dL, BMI ≥ 18 and ≤30.0 kg/m^2^. The subjects were given either Physta^®^ 100 mg, 200 mg or placebo daily for 12 weeks. The primary endpoints were changes in serum total and free testosterone levels. The secondary endpoints included changes in the level of sex hormone-binding globulin (SHBG), dihydroepiandrosterone (DHEA), glycated haemoglobin (HbA1c), insulin-like growth factor-1 (IGF-1), thyroid function tests (T3, T4, TSH and Free T3) and cortisol. Changes in Ageing Male Symptoms (AMS) score, Fatigue Severity Scale (FSS) score and muscle strength are other secondary endpoints. The safety of the intervention products was measured by complete blood count, lipid profile, liver and renal function tests.

**Results:**

There was a significant increase in the total testosterone levels at week 12 (*P* < 0.05) in the Physta^®^ 100 mg group and at weeks 4 (*P* < 0.05), 8 (*P* < 0.01) and 12 (*P* < 0.001) in the Physta^®^ 200 mg group compared to placebo. No significant between-group differences in free testosterone levels were observed but a significant within-group increase occurred at weeks 4 (*P* < 0.01), 8 (*P* < 0.001) and 12 (*P* < 0.001) in the Physta^®^100 mg group and at weeks 2 (*P* < 0.01), 4 (*P* < 0.01), 8 (*P* < 0.001) and 12 (*P* < 0.001) in the Physta^®^ 200 mg group. The AMS and FSS showed significant reduction (*P* < 0.001) in total scores at all time-points within- and between-group in both Physta^®^ groups. DHEA levels significantly increased (*P* < 0.05) within-group in both Physta^®^ groups from week 2 onwards. Cortisol levels significantly (*P* < 0.01) decreased in the Physta^®^ 200 mg group, while muscle strength significantly (*P* < 0.001) increased in both Physta^®^ groups at week 12 in the within-group comparison. There were no significant changes in SHBG. No safety related clinically relevant changes were observed.

**Conclusion:**

Supplementation of Physta^®^ at 200 mg was able to increase the serum total testosterone, reduce fatigue and improve the quality of life in ageing men within 2 weeks’ time.

**Trial registration:**

This clinical study has been registered in ctri.nic.in (CTRI/2019/03/017959).

## Popular scientific summary

- Lower free and total testosterone concentrations are significantly common in older population, and it is associated with a reduced quality of life.
- Physta^®^, *Eurycoma longifolia* water extract supplementation at 100 and 200 mg able to improve the total testosterone levels, reduced ageing symptoms, and fatigue as early as 4 and 2 weeks of supplementation, respectively.

Physiological functions gradually decline with advancing age; examples of changes in physiological functions during ageing are decline in the capacity of cellular protein synthesis, decrease in immune function, loss of muscle mass and strength, increase in fat mass and decrease in bone mineral density (1). These changes during ageing can be related to changes in endocrine activity (1, 2), such as menopause, andropause and somatopause, which arise due to decreased circulating hormones (1–4).

Various literatures from endocrinology, urology and gerontology suggest that a syndrome similar to the menopause exists in men, referred to as the andropause, male climacteric, viropause or low testosterone syndrome (5). Andropause is accompanied by the physical characteristic, sexual and emotional symptoms resulting from hormonal, psychological, situational and physical factors (6). Physical symptoms include weakness, fatigue, reduced muscle and bone mass, and impaired haematopoiesis. Sexual dysfunction involves oligospermia, diminished libido and impotence, whereas the psychological and emotional component may involve depression, anxiety, irritability, insomnia, memory impairment, and reduced cognitive function (5).

There is a gradual decrease in the serum total and free testosterone levels during andropause (4). Testosterone production in men is managed by the hypothalamic–pituitary–gonadal (HPG) axis. Gonadotropin-releasing hormone (GnRH) secreted from the hypothalamus will stimulate the pituitary gland to release luteinising hormone (LH) and follicle-stimulating hormone (FSH). LH will activate the testicular Leydig cells to produce testosterone (7). In healthy adult men, only 2–3% of testosterone exists as unbound and free form (8, 9), which is known as free testosterone (7). About 40–50% of testosterone is strongly bound to the sex hormone-binding globulin (SHBG), and the remaining testosterone is bound loosely to albumin (9). The free testosterone and the albumin testosterones are known as bioavailable testosterones, which are available for biological action. Lower free and total testosterone concentrations are significantly common in older population, and it is associated with a reduced quality of life (10).

Testosterone levels decline with age (2, 11–13) in the order of 100 ng/dL every decade (14) and affect the quality of life. Other than the low testosterone levels, ageing is associated with changes in various hormones, that is, DHEA, a precursor of testosterone, also declines more rapidly and significantly with ageing (3). SHBG, which is known for the binding affinity with testosterone, increases during ageing and leads to a decrease in the levels of free and bioavailable testosterones (15). Thyroid hormones, especially T4 and T3, might involve in energy metabolism (16), and both hormones are known to decrease during ageing in men (17).

A multitude of herbal products claims to potentially increase testosterone, of which *Eurycoma longifolia* (EL) has shown promising effects in increasing the testosterone concentrations (18). EL has been used as a traditional medicine for its aphrodisiac effects and treatment of intermittent fever (malaria) (19). The roots of EL are largely responsible for its biological activity due to the presence of peptides, alkaloids, quassinoids, diterpenoids, eurycomacoside, eurycolactone, laurylcolactone and eurycomalactone (20–22). It is also used as an adaptogen for vitality and energy (23). Supplementation of EL increases the restoration of testosterone levels gradually in human as it enhances the natural biological synthesis of testosterone (24–26). EL has demonstrated its aphrodisiac properties in animal model studies (27–29) and human clinical studies (30, 31).

Various EL extracts and EL-based products are available in market but studies to support the efficacy and safety of such products are limited. Physta^®^, a standardised water extract from the roots of EL, is widely studied in various animal models and clinical studies. Safety of Physta^®^ is well established based on acute, sub-acute and sub-chronic animal studies (32). The no observed adverse effect level (NOAEL) of Physta^®^ was concluded as more than 1,000 mg/kg orally. Subjects aged 28–70 years with late-onset hypogonadism (LOH) treated with 200 mg of Physta^®^ showed improved testosterone levels after 1 month (33). In another pilot study, physically active male and female participants aged 57–72 years supplemented with 400 mg Physta^®^ extract daily for 5 weeks showed significant increases in the free and total testosterone levels by the third week (26). However, this study involved a small number of subjects with a higher dose of Physta^®^, and evaluation was conducted only from week 3 onwards. No studies have demonstrated the effect of Physta^®^ at lower doses or over shorter supplementation duration in a male population aged 50–70 years. The objective of this study was to investigate the effect of two different doses of Physta^®^, that is, 100 and 200 mg of the standardized aqueous extract of EL root on the total and free testosterones, and the quality of life (QoL) in the older male subjects aged 50–70 years.

## Material and methods

The study was conducted in accordance with the good clinical practice (GCP), Declaration of Helsinki guidelines. The study was conducted at the Oriana Hospital, Varanasi, Uttar Pradesh, India, and Lokmanya Multi-Speciality Hospital, Pune, Maharashtra, India. The study was approved on 16 February 2019 by the Oriana Hospital Ethics Committee at Varanasi and on 6 May 2019 at Pune by the Independent Research Ethics Committee prior to the initiation of any study-related activities involving human subjects. Written informed consent was obtained from the volunteers before all the study procedures. The recruitment and follow-up took place from 27 March 2019 to 14 September 2019.

### Study design

This was a double-blind, placebo-controlled, multicentre, randomised, phase II clinical trial with a 12-week safety and efficacy monitoring period. The sample size was calculated by comparing the primary endpoint (changes in testosterone levels) between Physta^®^ and placebo groups with a two-sided two sample test. An average increase of 37.5 ng/dL in the testosterone level was obtained in the previous study for a combined extract of EL and *Polygonum minus* (30). In addition, a between-factor repeated-measure analysis of variance (ANOVA) with a level of significance (α) of 0.05 and power of 80% was considered to obtain the minimum increase of 37.5 ng/dL in the testosterone level between Physta^®^ and placebo groups. The enrolment ratio between the trial and control groups was set as 1, resulting in 35 subjects per group. As this is a three-arm study, sample size 105 per-protocol (PP) subjects were required.

Participants were required to make six visits to the clinical sites. On the first visit, inclusion/exclusion criteria, medical history, demographic, concomitant therapies and physical examination were reviewed; baseline vital signs were collected; and BMI was calculated. Blood samples were obtained for the assessment of safety profile (haematology and clinical chemistry) and study-specific markers, including testosterone (free and total), SHBG, DHEA, cortisol, IGF-1, thyroid function hormones (T3, T4, thyroid-stimulating hormone [TSH], Free T3), HbA1c and lipid profile. Subjects who met the inclusion criteria and none of the exclusion criteria were enrolled in the study, and written informed consents were obtained.

Subjects who were eligible were randomised on visit 2 (Week 0), physical examination and vital signs were performed, AMS (Ageing Male Symptoms) and FSS (Fatigue Severity Scale) questionnaires were administered to assess the symptoms of low testosterone; and the muscle strength was assessed using a back leg chest (BLC) dynamometer. Subjects were required to come for the follow-up visits on week 2, 4, 8 and 12. At each visit, vital signs, physical examination, concomitant medication therapy, treatment compliance and adverse events were reviewed; AMS and FSS questionnaires were administered and their scores were recorded; blood was collected for serum total and free testosterone, SHBG and DHEA measurements. Muscle strength test, safety and other study-specific lab tests were performed at screening/baseline and at the end of week 12. The investigational product was dispensed at week 0, 4 and 8 along with the subject dosing diary. Subjects were instructed to maintain the dosing diary to measure the treatment compliance throughout the study and were required to record concomitant therapies, adverse events (AEs) and serious adverse events (SAEs).

### Participants

The study recruited 105 participants from the internal database of the respective hospitals (study sites). Inclusion criteria were as follows: healthy volunteers in the age range 50 to 70 years with a BMI 18–30.0 kg/m^2^, and testosterone level <300 ng/dL. The volunteers were expected to be in a heterosexual relationship for at least 6 months prior to enrolment and willing to discontinue any other product which could boost testosterone during the study.

Exclusion criteria were as follows: participants were excluded if they had a history of prostate cancer and other type of malignancy, penile abnormalities, cardiovascular disease, uncontrolled hypertension/hypotension, uncontrolled diabetes, history of seizures, clinically significant chronic haematological disease, significant active peptic ulceration and any congenital brain injuries or mental illness. In total, 113 subjects were screened; of these, seven subjects did not meet the selection criteria, and the remaining 106 subjects were randomised into either Physta^®^ 100 mg, Physta^®^ 200 mg or placebo group.

### Investigational product

The investigational product was Physta^®^, a standardised water-soluble extract of EL roots, is commercially available from Biotropics Malaysia standardised with the following specification 0.8–1.5% eurycomanone, not less than 22% total protein, 30.0% total polysaccharide and 40.0% glycosaponin. The study products Physta^®^ 100 mg and Physta^®^ 200 mg were produced under the continuous quality of good manufacturing process (cGMP) requirements at Biotropics Malaysia Berhad, Shah Alam, Selangor, Malaysia. It was provided as transparent, hard gelatine capsules containing either 100 mg of Physta^®^ with 250 mg maltodextrin or 200 mg of Physta^®^ with 150 mg maltodextrin, respectively. Placebo was prepared as an identical capsule containing 280 mg of maltodextrin. The subjects were asked to take either Physta^®^100 mg, Physta^®^200 mg or placebo, once daily after breakfast starting from the day after the randomisation visit and continuing until week 12.

### Outcome measures

The primary outcome was the change in serum testosterone and free testosterone in Physta^®^ groups versus placebo at weeks 2, 4, 8 and 12, and within-group change from baseline to weeks 2, 4, 8 and 12 in Physta^®^ groups. The secondary objective was to compare the efficacy of Physta^®^ to placebo from baseline to weeks 2, 4, 8 and 12 in terms of change in QoL using AMS and FSS questionnaires. AMS is a validated questionnaire, widely used to evaluate the severity of AMS in older men (34). The AMS questionnaire includes 17 questions comprising of three subscales (psychological, somatic and sexual symptoms). A total score ≥27 has been defined as the deficiency in androgen level, and a significant reduction in the AMS score was expected at end of the study as it will indicate that the supplementation ameliorates the ageing symptoms (35). FSS is a 9-item validated questionnaire that investigates the severity of fatigue, and higher score is expected when the fatigue severity is greater (36).

Additional secondary efficacy endpoints were the changes in SHBG, DHEA, HbA1c, IGF-1, thyroid function test (T3, T4, TSH and Free T3) and cortisol levels. Another secondary endpoint was the muscle strength, and it was assessed using the BLC dynamometer test at baseline and week 12. Safety and tolerability of the investigational products were assessed through changes in the complete blood count (CBC), liver function test (LFT), renal function test (RFT) and lipid profile from baseline to week 12; all AEs, SAEs and vital signs were measured at all visits.

### Compliance

The dispensed study dosing diaries were returned to the study sites, and participants who were non-compliant with their diaries were reminded of their obligations regarding the study compliance. Compliance was assessed by comparing the number of returned unused product versus the number of dosages expected to have been taken during each visit period.

### Statistical analysis

The PP analysis included subjects who consumed at least 80% of either product, did not have any major protocol deviation and have completed all study visits and procedures related to the measurement of the primary endpoints. Statistical analysis was performed using the Graph Pad Prism 8, and the normal distribution of the data was assessed by the Kolmogorov–Smirnov test and expressed as mean ± standard deviation (SD). Descriptive statistics were calculated, and statistical comparisons were performed using the ANOVA, followed by the Dunnett’s test for within-group comparisons from baseline to week 2, 4, 8 and 12 for total and free testosterone, SHBG, DHEA, BMI, AMS, FSS and vital signs. Paired *t*-test was performed to determine within-group differences from baseline to week 12 for HbA1C, IGF-1, thyroid function test (T3, T4, Free T3 and TSH), cortisol, muscle tone, CBC, LFT, RFT and lipid profile. Statistical comparisons between groups at each time point of the treatment group versus placebo were determined using ANOVA followed by Dunnett’s test for all parameters.

### Ethics approval and consent to participate

The study protocol was approved by the Oriana Hospital Ethics Committee of Varanasi, Uttar Pradesh, India (ECR/1003/Inst/UP/2017), and Independent Research Ethics Committee, Pune, Maharashtra, India (ECR/232/Indt/MH/2015/RR-2018). All study participants gave written informed consent to take part in the clinical study.

## Results

### Participant demographics

**Fig. 1:**
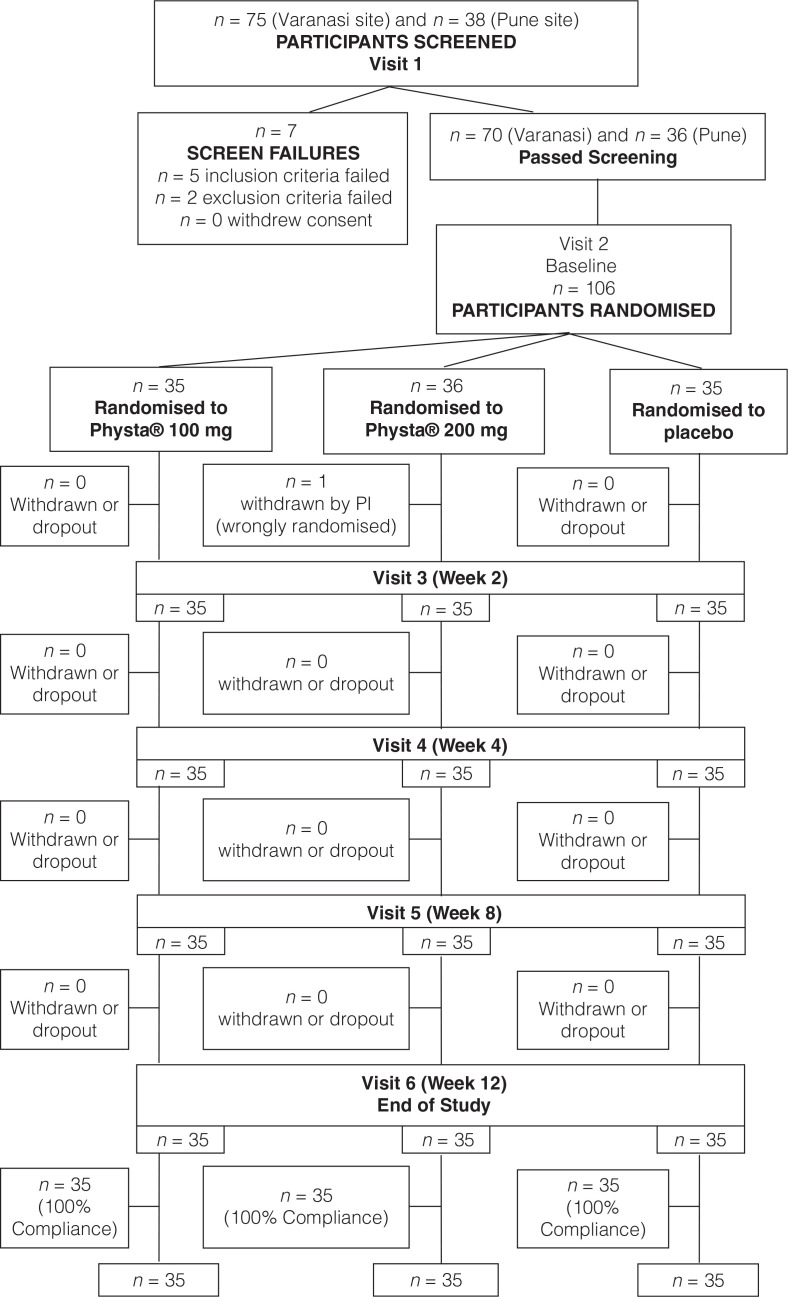
Randomisation and treatment schedule flowchart.

A total of 113 subjects were screened at two study sites, 106 were enrolled and one subject who found to be enrolled into wrong group was excluded after randomisation (visit 2); 105 subjects completed the study with 35 subjects each in Physta^®^ 100 mg, Physta^®^ 200 mg and placebo group. Demographic characteristics indicated that the study population was homogeneous, with no statistically significant differences between the groups at baseline (Table 1). The mean compliance for Physta^®^ 100 mg, Physta^®^ 200 mg and placebo groups was 100% (Fig. 1).

**Table 1:** Baseline characteristics of vitals and anthropometric measures of all subjects

Parameter	Physta 100 mg	*P*-value *Physta 100 mg vs. Placebo	Physta 200 mg	*P*-value *Physta 200 mg vs. Placebo	Placebo
Age^a^ (years)	56.94 ± 4.61	0.94	57.03 ± 5.09	0.92	56.60 ± 5.22
Height^a^ (cm)	163.5 ± 6.26	0.32	163.8 ± 3.86	0.08	161.4 ± 6.85
Weight^a^ (kg)	60.93 ± 7.14	>0.99	60.47 ± 5.40	0.97	60.76 ± 6.60
Temperature^a^ (ºC)	37.04 ± 0.19	0.75	36.98 ± 0.14	0.63	37.01 ± 0.21
Pulse^a^ (beats/min)	78.54 ± 6.20	0.73	79.34 ± 7.46	0.43	77.83 ± 4.96
Systolic blood pressure^b^ (mmHG)	123.60 ± 6.82	0.32	123.80 ± 6.30	0.41	125.37 ± 6.54
Diastolic blood pressure^b^ (mmHG)	82.00 ± 4.80	>0.99	82.31 ± 4.95	0.98	82.11 ± 4.15

Data stated as mean ± SD, *n* = 35 in each group, *P* < 0.05 considered statistically significant.

* Between-group analysis: Physta 100 mg and Physta 200 mg compared with placebo.

a Analysed by one-way ANOVA followed by Dunnett’s multiple comparisons test; ^b^Analysed by two-way ANOVA followed by Dunnett’s multiple comparisons test.

### Primary endpoints

There was a significant increase (*P* < 0.05) in the total testosterone for Physta^®^ 100 mg at week 12; for the Physta^®^ 200 mg group, significant increase was seen at week 4 (*P* < 0.05) and maintained until week 12 (*P* < 0.001) compared to placebo. The within-group analysis of Physta^®^100 mg showed a significant increase (*P* < 0.05) in the total testosterone from week 8 to 12 (*P* < 0.01), whereas the supplementation with Physta^®^ 200 mg showed a gradual and higher significant increase (*P* < 0.001) at every time point compared to baseline (Table 2).

**Table 2:** Total and free testosterone levels by group and visit

Parameters	Group	Baseline	*P*-value Placebo vs. Physta^®^	Week 2	*P*-value Placebo vs. Physta^®^	Week 4	*P*-value Placebo vs. Physta^®^	Week 8	*P*-value Placebo vs. Physta^®^	Week 12	*P*-value Placebo vs. Physta^®^
Total testosterone (ng/dl)	Physta^®^ 100 mg	187.3 ± 46.4	0.86	190.3 ± 50.0	0.89	194.9 ± 51.2	0.60	198.6 ± 50.6^#^	0.26	203.8 ± 54.6^##^	0.04^*^
	Physta^®^ 200 mg	200.5 ± 46.4	0.14	207.9 ± 45.0^###^	0.06	215.4± 48.1^###^	0.01^*^	218.9± 46.7^###^	0.002^**^	225.0± 49.8^###^	<0.001^***^
	Placebo	183.0 ± 37.8		186.5 ± 37.5		186.7 ± 36.5		185.0 ± 36.4		177.9 ± 43.7	
Free testosterone (pg/ml)	Physta^®^ 100 mg	16.78 ± 12.53	>0.99	16.98 ± 12.69	0.85	17.17± 12.70^##^	0.96	17.41 ± 12.77^###^	>0.99	17.63 ± 12.73^###^	>0.99
	Physta^®^ 200 mg	14.09 ± 10.82	0.15	15.55 ± 12.43^##^	0.35	15.91± 12.60^##^	0.50	16.25 ± 12.66^###^	0.69	16.47 ± 12.84^###^	0.71
	Placebo	16.87 ± 14.69		17.45 ± 15.47		17.39 ± 15.44		17.31 ± 15.35		17.56 ± 15.71	

Data stated as mean ± SD, *n* = 35 in each group.

Significant between-group analysis: ^*^P < 0.05, ^**^P < 0.01, ^***^P < 0.001; Between-group analysis: Physta^®^ 100 mg and Physta^®^ 200 mg compared to placebo at each time-point by ANOVA followed by Dunnett’s multiple comparisons test.

Significant within-group analysis: ^#^*P* < 0.05, ^##^*P* < 0.01, ^###^*P* < 0.001; Baseline value compared to every other time point for Physta^®^ 100 mg, 200 mg and placebo groups by ANOVA followed by Dunnett’s multiple comparisons test.

There were no significant between-group differences in the free testosterone levels for Physta^®^ 100 and 200 mg compared to placebo at all-time points. The within-group differences in the free testosterone levels of the subjects supplemented with Physta^®^ 100 mg showed a significant increase (*P* < 0.01) from week 4 to 12 (*P* < 0.001), whereas there was a significant increase at all-time points for Physta^®^ 200 mg compared to baseline (Table 2).

### Secondary efficacy assessment

The AMS total scores showed a significant reduction (*P* < 0.001) at each time point after baseline in the Physta^®^ 100 mg and Physta^®^ 200 mg groups compared to placebo and baseline. Similar results were observed for FSS scores where a significant (*P* < 0.001) decrease was observed in the between-group analysis when compared to placebo from week 2 onwards and within-groups when compared to baseline (Table 3). No significant within-group changes were observed in the placebo group for AMS and FSS scores. These results indicate that the supplementation of Physta^®^ was able to reduce the symptoms of ageing and fatigue.

**Table 3:** AMS and FSS score, SHBG, DHEA and BMI levels by group and visit

Parameters	Group	Baseline	*P*-value Placebo vs. Physta^®^	Week 2	*P*-value Placebo vs. Physta^®^	Week 4	*P*-value Placebo vs. Physta^®^	Week 8	*P*-value Placebo vs. Physta^®^	Week 12	*P*-value Placebo vs. Physta^®^
AMS	Physta^®^ 100 mg	58.91 ± 12.26	>0.99	52.63± 11.7^###^	<0.001^***^	47.49± 11.88^###^	<0.001^***^	40.37± 9.801^###^	<0.001^***^	35.43± 10.93^###^	<0.001^***^
	Physta^®^ 200 mg	58.34 ± 11.58	0.72	47.77± 6.860^###^	<0.001^***^	39.11± 4.057^###^	<0.001^***^	31.91± 3.641^###^	<0.001^***^	26.71± 8.986^###^	<0.001^***^
	Placebo	58.94 ± 12.01		57.71 ± 12.64		57.69 ± 13.89		57.11 ± 13.58	0.06	58.06 ± 13.22	
FSS	Physta^®^ 100 mg	57.54 ± 2.43	0.47	52.71± 5.60^###^	<0.001^***^	48.20± 5.49^###^	<0.001^***^	40.31± 6.34^###^	<0.001^***^	33.60 ± 5.09^###^	<0.001^***^
	Physta^®^ 200 mg	57.86 ± 2.568	>0.99	50.34 ± 8.842^###^	<0.001^***^	43.34± 9.159^###^	<0.001^***^	35.66± 10.98^###^	<0.001^***^	29.17 ± 11.34^###^	<0.001^***^
	Placebo	57.86 ± 2.30		57.83 ± 3.20		58.40 ± 3.35		58.26 ± 2.74		58.54 ± 4.60	
SHBG (nmol/L)	Physta^®^ 100 mg	29.11 ± 15.67	>0.70	29.27 ± 14.81	0.65	29.32 ± 14.81	0.65	29.05 ± 14.95	0.69	29.09 ± 14.85	0.74
	Physta^®^ 200 mg	27.32 ± 12.90	>0.99	28.23 ± 12.94	0.86	28.48 ± 13.26	0.81	28.69 ± 13.44	0.72	8.20 ± 13.01	0.90
	Placebo	27.12 ± 10.55		27.12 ± 10.52		27.16 ± 10.56		27.03 ± 10.58		27.31 ± 10.14	
DHEA (ng/ml)	Physta^®^ 100 mg	2.802 ± 0.832	0.55	2.888 ± 0.818^#^	0.55	2.992 ± 0.829^##^	0.73	3.063 ± 0.851^###^	0.87	3.105 ± 0.864^###^	0.82
	Physta^®^ 200 mg	3.233 ± 1.603	>0.99	3.388 ± 1.60^#^	0.95	3.489 ± 1.579^###^	0.82	3.502± 1.599^###^	0.77	3.553 ± 1.619^##^	0.79
	Placebo	3.199 ± 2.295		3.279 ± 2.258		3.269 ± 2.243		3.248 ± 2.245		3.316 ± 2.177	
BMI (kg/m^2^)	Physta^®^ 100 mg	23.37 ± 2.34	0.38	23.38 ± 2.24	0.40	23.39 ± 2.25	0.35	23.36 ± 2.29	0.33	23.31 ± 2.25	0.56
	Physta^®^ 200 mg	22.79 ± 2.06	0.19	22.77 ± 2.05	0.14	22.75 ± 2.05	0.13	22.76 ± 2.000	0.13	22.88 ± 1.94	0.20
	Placebo	22.55 ± 1.978		22.52 ± 1.826		22.54 ± 1.827		22.52 ± 1.837		22.56 ± 1.807	

Data stated as mean ± SD, *n* = 35 in each group, *P* < 0.05 considered statistically significant.

Significant between-group analysis: ***P < 0.001; Between-group analysis: Physta^®^ 100 mg and Physta^®^ 200 mg compared to placebo at each time point by ANOVA followed by Dunnett’s multiple comparisons test.

Significant within-group analysis: ^#^*P* < 0.05, ^##^*P* < 0.01, ^###^*P* < 0.001; Baseline value compared to every other time point for Physta^®^ 100 mg, 200 mg and placebo groups by ANOVA followed by Dunnett’s multiple comparisons test.

There were no significant changes within-group and between-groups in SHBG concentration in both treatment groups. No significant between-group changes were observed with Physta^®^ 100 mg and Physta^®^ 200 mg in DHEA level compared to placebo but there was a significant (*P* < 0.05) increase in DHEA concentration in both Physta^®^ groups at weeks 2, 4, 8 and 12 compared to their respective baseline values (Table 3).

The secondary efficacy parameters HbA1c, IGF-1, thyroid function test (T3, T4, Free T3 and TSH), cortisol and muscle strength were evaluated at baseline and week 12. There was no significant between-group differences in HbA1c for Physta^®^ groups compared to placebo, but a significant (*P* < 0.05 and *P* < 0.01) within-group decrease in HbA1c was seen for both Physta^®^ 200 mg and placebo groups, respectively. There were no significant differences between groups in IGF-1 at baseline and week 12 in both Physta^®^ groups compared to placebo, but in Physta^®^ 100 mg group, IGF-1 levels significantly decreased (*P* < 0.05) at week 12 compared to baseline (Table 4).

**Table 4:** Secondary outcome variables by group and visit

Parameter	Group	Baseline	*P*-value *Placebo vs. Physta^®^	Week 12	*P*-value *Placebo vs. Physta^®^
HbA1c (%)	Physta^®^100 mg	5.60 ± 0.76	0.52	5.44 ± 0.60	>0.99
	Physta^®^200 mg	5.84 ± 0.60	0.95	5.57 ± 0.50^#^	0.53
	Placebo	5.79 ± 0.80		5.43 ± 0.56^##^	
IGF-1 (ng/ml)	Physta^®^100 mg	138.0 ± 34.2	0.23	132.9 ± 34.8^#^	0.32
	Physta^®^200 mg	135.4 ± 34.3	0.39	129.7 ± 32.6	0.53
	Placebo	126.2 ± 30.7		122.6 ± 33.4	
T3 (nmol/l)	Physta^®^100 mg	1.779 ± 0.408	0.94	1.950 ± 0.490^#^	0.05^*^
	Physta^®^200 mg	1.873 ± 0.570	0.47	1.803 ± 0.565	0.57
	Placebo	1.753 ± 0.604		1.709 ± 0.532	
T4 (nmol/l)	Physta^®^100 mg	102.8 ± 20.9	0.68	97.5 ± 20.0^#^	0.67
	Physta^®^200 mg	104.5 ± 25.6	0.41	103.0 ± 24.2	>0.99
	Placebo	99.0 ± 25.4		102.4 ± 26.7	
TSH (uIU/ml)	Physta^®^100 mg	2.992 ± 1.316	0.72	2.833 ± 1.251	0.79
	Physta^®^200 mg	2.768 ± 1.311	0.98	2.700 ± 1.102	0.99
	Placebo	2.817 ± 1.455		2.670 ± 1.238	
Free T3 (pmol/l)	Physta^®^100 mg	5.291 ± 0.615	0.009^**^	5.431 ± 0.820^#^	0.02^*^
	Physta^®^200 mg	5.046 ± 0.891	0.32	5.072 ± 0.9228	0.57
	Placebo	4.827 ± 0.657		4.871 ± 0.8979	
Cortisol (nmol/l)	Physta^®^100 mg	292.6 ± 146.7	0.92	280.6 ± 153.2	0.69
	Physta^®^200 mg	284.6 ± 156.8	>0.99	252.8 ± 163.0^##^	0.80
	Placebo	284.6 ± 155.2		266.1 ± 163.6	
Muscle Strength (Kg)	Physta^®^100 mg	59.12 ± 6.35	>0.99	60.67 ± 5.60^###^	0.52
	Physta^®^200 mg	60.07 ± 6.47	0.72	63.06 ± 7.27^###^	0.01^*^
	Placebo	59.19 ± 6.96		59.17 ± 6.84	

Data stated as mean ± SD, *n* = 35 in each group.

Significant between-group analysis: ^*^P < 0.05, ^**^P < 0.01; Between-group analysis: Physta^®^ 100 mg and Physta^®^ 200 mg compared to placebo at each time point by ANOVA followed by Dunnett’s multiple comparisons test.

Significant within-group analysis: ^#^*P* < 0.05, ^##^*P* < 0.01, ^###^*P* < 0.001; Baseline value compared to every other time point for Physta^®^ 100 mg, 200 mg and placebo groups by paired *t*-test.

In the Physta^®^ 200 mg group, no significant changes were recorded for thyroid function hormones in between-group compared to placebo and within-group when compared to baseline. Similar results were seen in Physta^®^ 100 mg group, except some significant changes noted in T3, free T3 and T4 levels. There was a significant (*P* < 0.05) increase in both T3 and free T3 levels in the Physta^®^ 100 mg group compared to placebo and baseline at week 12; meanwhile, T4 levels were found to be significantly (*P* < 0.05) decreased at week 12 compared with baseline (Table 4).

There were no significant differences in the cortisol level in the Physta^®^ groups at baseline and week 12 compared to placebo but a significant (*P* < 0.01) decrease from baseline is seen at week 12 in the Physta^®^ 200 mg group. Based on the between-group analysis, a significant (*P* < 0.05) increase in the muscle strength was observed at week 12 in subjects supplemented with Physta^®^ 200 mg but no significant difference was seen in Physta^®^100 mg group. The within-group analysis revealed a significant (*P* < 0.001) increase in both Physta^®^ 100 mg and Physta^®^ 200 mg groups for muscle strength compared to their baseline values, respectively (Table 4).

### Safety assessment

There were no significant within-group or between-group differences in the vital signs, including BMI throughout the study for both Physta^®^ groups. No significant differences were observed in any haematology parameters in both treatment groups compared to placebo, except for mean platelet volume (MPV) which significantly lower at week 12 (*P* < 0.01) in the Physta^®^200 mg group. No significant changes were found in any parameters of RFT, LFT and lipid profile in both treatment groups compared to placebo. Significant (*P* < 0.05) within-group changes were observed in MPV, eosinophil, monocytes, basophil and creatinine levels in Physta^®^ and placebo groups. Some significant differences (*P* < 0.05) were observed in the parameters as platelet, haematocrit, mean corpuscular volume (MCV), lymphocytes and bilirubin compared to baseline values either in Physta^®^ or placebo group (Table 5). All significant changes in the safety parameters were within a normal clinical reference range (Table 5).

**Table 5:** Safety outcome variables by group and visit

Parameter	Reference value	Group	Baseline	*P*-value ^*^Placebo vs. Physta^®^	Week 12	*P*-value ^*^Placebo vs. Physta^®^
Haemoglobin (gm/dl)	13–16	Physta^®^100 mg	14.04 ± 1.68	0.12	14.22 ± 1.33	0.12
		Physta^®^200 mg	13.87 ± 1.85	0.40	14.11 ± 1.40	0.30
		Placebo	13.43 ± 1.76		13.67 ± 1.64	
Red blood cell (million/Cu.m)	4–6	Physta^®^100 mg	4.932 ± 0.755	0.08	4.850 ± 0.724	0.46
		Physta^®^200 mg	4.684 ± 0.796	0.97	4.733 ± 0.718	0.95
		Placebo	4.656 ± 0.640		4.695 ± 0.689	
White Blood cell (million/Cu.mm)	4,000–10,000	Physta^®^100 mg	8.063 ± 1.949	0.33	7.639 ± 1.989	0.92
		Physta^®^200 mg	7.113 ± 1.539	0.59	7.107 ± 1.721	0.87
		Placebo	7.557 ± 2.021		7.259 ± 2.007	
Platelet count (Lac/Cu.mm)	1.5–4.5	Physta^®^100 mg	1.998 ± 0.904	0.56	2.223 ± 0.815^##^	>0.99
		Physta^®^200 mg	2.005 ± 0.744	0.48	2.215 ± 0.698^#^	>0.99
		Placebo	2.121 ± 0.747		2.219 ± 0.758	
Haematocrit (%)	37–54	Physta^®^100 mg	42.35 ± 4.70	0.07	41.53 ± 4.017^#^	0.57
		Physta^®^200 mg	41.84 ± 5.28	0.28	41.69 ± 3.935	0.45
		Placebo	40.34 ± 4.87		40.78 ± 4.629	
Mean corpuscular volume (fL)	85–95	Physta^®^100 mg	89.69 ± 5.51	0.25	89.53 ± 4.68	>0.99
		Physta^®^200 mg	90.89 ± 9.04	0.15	89.41 ± 4.83	0.97
		Placebo	87.09 ± 8.92		89.62 ± 4.34^#^	
Mean corpuscular haemoglobin (pg)	28–32	Physta^®^100 mg	28.67 ± 2.01	0.99	30.08 ± 2.84^##^	0.97
		Physta^®^200 mg	29.68 ± 3.42	0.34	30.53 ± 2.83	0.71
		Placebo	28.60 ± 3.23		30.17 ± 1.71^##^	
Mean corpuscular haemoglobin concentration (gm/dl)	32–34	Physta^®^100 mg	33.74 ± 1.50	0.88	33.09 ± 1.56^##^	0.47
		Physta^®^200 mg	33.57 ± 1.66	0.96	33.53 ± 1.60	0.94
		Placebo	33.64 ± 1.44		33.45 ± 1.44	
Mean platelet volume (fL)	6.6–12	Physta^®^100 mg	12.49 ± 2.99	0.01^*^	9.860 ± 1.39^###^	0.69
		Physta^®^200 mg	12.01 ± 3.11	0.38	9.002 ± 1.35^###^	0.002^**^
		Placebo	11.45 ± 2.96		10.12 ± 1.44^#^	
Neutrophil (%)	50–70	Physta^®^100 mg	63.49 ± 9.42	>0.99	63.91 ± 5.15	>0.99
		Physta^®^200 mg	61.43 ± 7.08	0.43	63.29 ± 4.34	0.68
		Placebo	63.54 ± 9.00		64.03 ± 4.49	
Eosinophil (%)	1–6	Physta^®^100 mg	4.714 ± 3.670	0.52	2.371 ± 1.35^##^	>0.99
		Physta^®^200 mg	3.686 ± 2.53	0.75	2.457 ± 1.27^#^	0.91
		Placebo	4.029 ± 1.98		2.371 ± 1.22^###^	
Lymphocytes (%)	25–40	Physta^®^100 mg	27.29 ± 7.17	0.95	30.86 ± 4.60^##^	0.96
		Physta^®^200 mg	30.43 ± 6.76	0.25	31.40 ± 4.13	0.94
		Placebo	27.80 ± 7.96		31.11^*^± 3.81^#^	
Monocytes (%)	2–10	Physta^®^100 mg	4.45 ± 2.214	>0.99	2.629 ± 1.629^###^	0.97
		Physta^®^200 mg	4.457 ± 2.187	0.99	2.800 ± 1.587^###^	0.74
		Placebo	4.486 ± 2.147		2.543 ± 1.704^###^	
Basophil count (%)		Physta^®^100 mg	0.234 ± 0.322	>0.99	0.000 ± 0.000^###^	-
	0–2	Physta^®^200 mg	0.183 ± 0.279	0.40	0.000 ± 0.000^###^	-
		Placebo	0.236 ± .2909		0.000 ± 0.000^###^	
Serum glutamic-oxaloacetic transaminase (IU/L)	<50	Physta^®^100 mg	34.12 ± 9.070	0.88	34.20 ± 9.921	0.97
		Physta^®^200 mg	34.17 ± 9.673	0.89	34.25 ± 9.737	0.98
		Placebo	33.11 ± 11.99		34.61 ± 8.930	
Serum glutamic pyruvic transaminase (IU/L)	<50	Physta^®^100 mg	35.43 ± 15.29	0.89	35.90 ± 9.87	>0.99
		Physta^®^200 mg	35.65 ± 14.72	0.81	35.78 ± 9.56	0.98
		Placebo	33.73 ± 18.51		36.15 ± 10.89	
Bilirubin Total (mg/dL)	0.3–1.2	Physta^®^100 mg	0.695 ± 0.272	>0.99	0.663 ± 0.156	0.96
		Physta^®^200 mg	0.731 ± 0.218	0.84	0.646 ± 0.167^#^	0.74
		Placebo	0.702 ± 0.228		0.674 ± 0.174	
Uric acid (mg/dL)	3.4–7.0	Physta^®^100 mg	5.692 ± 1.355	0.55	5.035 ± 0.984^##^	0.88
		Physta^®^200 mg	5.594 ± 1.424	0.67	5.198 ± 1.128	0.52
		Placebo	5.343 ± 1.344		4.931 ± 1.015^#^	
Blood urea nitrogen (mg/dL)	5.0–20	Physta^®^100 mg	12.33 ± 3.36	0.72	11.58 ± 3.12	0.26
		Physta^®^200 mg	11.41 ± 2.84	0.64	11.32 ± 3.29	0.13
		Placebo	11.92 ± 2.92		12.40 ± 4.07	
Creatinine (mg/dL)	0.6–1.4	Physta^®^100 mg	0.911 ± 0.145	0.84	0.820 ± 0.150^##^	0.55
		Physta^®^200 mg	0.871 ± 0.165	0.74	0.827 ± 0.1333^#^	0.75
		Placebo	0.894 ± 0.152		0.838 ± 0.1122^#^	
Triglycerides (mg/dL)	<200	Physta^®^100 mg	151.3 ± 75.9	>0.99	141.1 ± 55.8	0.96
		Physta^®^200 mg	159.1 ± 82.3	0.80	157.5 ± 51.2	0.22
		Placebo	150.0 ± 81.5		137.8 ± 59.1	
Total Cholesterol (mg/dL)	130–220	Physta^®^100 mg	167.2 ± 27.6	0.92	163.9 ± 31.8	0.99
		Physta^®^200 mg	164.0 ±27.5	0.65	163.2 ± 32.3	>0.99
		Placebo	169.4 ± 30.0		162.9 ± 34.8	
High density lipid (mg/dL)	35–60	Physta^®^100 mg	42.87 ± 6.86	0.41	42.46 ± 7.97	0.14
		Physta^®^200 mg	42.17 ± 8.49	0.28	43.68 ± 12.81	0.84
		Placebo	44.49 ± 6.67		44.81 ± 8.34	
Low density lipid (mg/dL)	60–130	Physta^®^100 mg	103.2 ± 23.44	>0.99	97.55 ± 26.01	0.83
		Physta^®^200 mg	97.35 ± 28.06	0.55	94.81 ± 30.81	0.58
		Placebo	102.8 ± 24.57		100.8 ± 31.57	
Very low density lipid (mg/dL)	<35	Physta^®^100 mg	30.36 ± 15.13	>0.99	29.49 ± 11.47	0.64
		Physta^®^200 mg	31.10 ± 16.75	0.93	31.63 ± 14.93	0.27
		Placebo	30.11 ± 16.27		28.09 ± 10.13	
LDL/HDL ratio	<3.0	Physta^®^100 mg	2.475 ± 0.830	0.48	2.396 ± 0.934	0.94
		Physta^®^200 mg	2.472 ± 0.934	0.43	2.296 ± 1.006	0.98
		Placebo	2.262 ± 0.802		2.334 ± 0.936	
Cholesterol Total/HDL ratio	<5.0	Physta^®^100 mg	3.993 ± 1.072	0.23	3.631 ± 1.039	0.75
		Physta^®^200 mg	3.898 ± 1.292	0.39	3.945 ± 1.322	0.81
		Placebo	3.589 ± 1.137		3.779 ± 1.126	

Data stated as mean ± SD, *n* = 35 in each group.

Significant between-group analysis: ^*^P < 0.05, ^**^P < 0.01; Between-group analysis: Physta^®^ 100 mg and Physta^®^ 200 mg compared to placebo at each time-point by ANOVA followed by Dunnett’s multiple comparisons test.

Significant within-group analysis: ^#^*P* < 0.05, ^##^*P* < 0.01, ^###^*P* < 0.001; Baseline value compared to every other time point for Physta^®^ 100 mg, 200 mg and placebo groups by paired *t*-test.

A total of eight AEs occurred during the study but there were no SAEs. The most common AEs are gastrointestinal symptoms like constipation, dyspepsia, abdominal pain, flatulence and abdominal discomfort which accounted for three subjects in the Physta^®^ 100 mg and three subjects in the Physta^®^ 200 mg group. There was one case of itching in the Physta^®^ 200 mg group and one case of fever in the placebo group. None of the AEs was categorised as ‘likely related’ or ‘related’ to investigational products. None of the participants required medical treatment or hospitalisation, and all AEs were resolved by the end of the study.

## Discussion

The study demonstrated a significant increase in total testosterone in both Physta^®^ groups compared to placebo and baseline, in line with a previous study of patients suffering from LOH, which showed a significant improvement in serum testosterone after 1-month supplementation with the same Physta^®^ extract (33). Another study investigated the ergogenic effect of Physta^®^ in elderly people and found Physta^®^ as a potential herbal supplement for physically active males and females in the age range of 57–72 years (26). In the same study, a significant increase in the total and free testosterone concentrations was observed with a supplementation of 400 mg/day Physta^®^ for 5 weeks (26). Both past studies discussed above (26, 33) treated the subjects with 200 and 400 mg of Physta^®^, and improvement in the testosterone concentration was observed after 1 month and 5 weeks of Physta^®^ treatment, respectively. However, the current study demonstrated the efficacy of Physta^®^ in increasing the testosterone concentration as early as 2 weeks of treatment at lower doses of 200 mg for the older subjects with a subsequent increase at 4, 6, 8 and 12 weeks. In this study, subjects with low testosterone (<300 ng/dL) were recruited, and therefore, the supplementation of Physta^®^ at lower dosages increases the testosterone level significantly, especially in an ageing population. In some studies, the supplementation of EL did not statistically improve the testosterone levels, probably due to the healthy and younger adult populations used in these studies (31). This further affirms EL as an adaptogenic plant that modulates healthy hormonal levels depending on the individuals’ existing health (37).

There are multiple underlying mechanisms related to the increase in testosterone after EL supplementation. Physta^®^, a water extract of EL, contains a 4.2 kDa peptide that can increase the synthesis of testosterone in Leydig cells (38). In a more recent study, EL root extract induced the testosterone synthesis along with an increase in LH and FSH but decreased the oestrogen level (39). This provided evidence that EL root extract may potentially down-regulate the oestrogen-mediated feedback effect on LH and FSH secretion in the HPG axis (39). Another study suggested that eurycomanone, the major quassinoid found in the EL root, significantly increased the testosterone production in a dose-dependent manner (40). Eurycomanone inhibits phosphodiesterase and aromatase, thus inhibiting the conversion of testosterone to oestrogen, leading to the enhanced production of testosterone by the Leydig cell explants (39). Physta^®^ is standardised to contain 0.8–1.5% eurycomanone, which may be responsible for the increase in testosterone concentration.

The decrease in testosterone level is accompanied by an increase in SHBG that reduces the levels of free and bioavailable testosterones (11–14). Even though there was no significant change in the levels of SHBG, significant increase in free testosterone levels in both treatment groups was seen at within-group level analysis. This could mean that Physta^®^ may reduce the binding affinity of SHBG towards testosterone, thereby increasing the free testosterone levels in both treatment groups without altering SHBG levels.

The low testosterone levels during ageing in men cause fatigue, decreased muscle mass and strength, increased body fat, and increased complaints and symptoms of ageing. The cumulative effect of these factors impairs the QoL of older men (41). This study investigated the effect of Physta^®^ on QoL by assessing the ageing symptoms and fatigue severity; the AMS scale measures the treatment effect on a full range of severity of complaints of ageing (42). The FSS questionnaire was employed to assess fatigue, including how fatigue affects motivation, exercise, physical functioning, carrying out duties, work and social life (43). The AMS and FSS data showed a significant between-group and within-group score reduction in the Physta^®^100 and 200 mg treatment groups with no change in the placebo group. In this study, as early as 2 weeks after the initiation of Physta^®^ supplementation, the FSS as well as AMS score decreased significantly and declined further at the following visits. After 12 weeks of treatment, the decrease in FSS and AMS score suggested that Physta^®^ supplementation was effective in improving the severity of symptoms related to the QoL in ageing men with low testosterone levels. The decrease in AMS score could be directly related to an increase in the testosterone level, as previous studies related to testosterone supplementation have proven to reduce AMS score (42).

A previous study that treated hypogonadal men with testosterone gel revealed that testosterone administration substantially improved fatigue in the subjects (44). The effect of Physta^®^ in the reduction of fatigue may be related to an increase in the total and free testosterone levels. EL has demonstrated the ability to reduce the recovery time from fatigue in athletes (45) and mood-boosting effects in moderately stressed subject (25) in past studies. The decrease in fatigue and mood disturbance may have contributed to the overall well-being measured through the AMS and FSS scores. The improvement seen in AMS and FSS scores aligned with an increase in the testosterone levels, as all these improvements were reported 2 weeks after the initiation of Physta^®^.

Ageing is associated with the decreased production of the adrenal androgens DHEA and DHEA sulphate (DS), paralleling that of the growth hormone-insulin-like growth factor-I (GH-IGF-I) axis (46), resulting in andropause (2). EL root extract contains bioactive complex peptides, also known as eurypeptides, which stimulates DHEA and then acts on androgen receptors to initiate the conversion of androstenedione to testosterone (33). This study further establishes that Physta^®^ significantly increased the DHEA levels, possibly because Physta^®^ contains 22–45% total protein and, in particular, the peptide that triggers testosterone production whereby DHEA is a precursor.

Testosterone plays a major role in maintaining the glycaemic control (46), with type 2 diabetes mellitus (DM2) reducing the biosynthesis of testosterone, and vice versa (47, 48). Since the testosterone level in subjects treated with Physta^®^ 200 mg group increased significantly, the reduction in HbA1c levels could be related to an increase in the testosterone level, as supported by previous studies which demonstrated that testosterone replacement therapy (TRT) can reduce HbA1c levels in subjects with testosterone deficiency and DM2 (49–52). A significant reduction in HbA1c was also observed in placebo group at the end of week 12 compared to baseline. Physical activity and diet could alter HbA1c values (53), as the participants in this study were not limited to any specific exercise regime or diet throughout the study period; there are possibilities that significant changes in the HbA1c can be caused by these factors. This further explains why no significant difference was observed within-group in HbA1c levels. Even though a significant reduction in HbA1c was observed in the placebo group compared to baseline, the significant reduction of HbA1c in Physta^®^ 200 mg group cannot be neglected, and it should be further evaluated in future studies.

The other important change that occurs in elderly men is age-related thyroid dysfunction. During ageing, the skeletal muscle decreases in mass and quality, and thyroid hormones, especially T3 and T4, might play an important role in this process (54). In the skeletal muscle, T4 is converted to T3 by deiodinase 2, and T3 is the main thyroid hormone that directly regulates gene expression in the nucleus (55); therefore, serum free T3 levels might have a more direct association with skeletal muscle function and regeneration. Low T3 syndrome commonly occurs in aged population, and this population was found to have lower physical performance (56). Physta^®^ supplementation of 100 mg could increase the conversion of T4 to T3, which could be related to significant improvement in the muscle strength observed in both Physta^®^ groups. Hypothyroidism may decrease the total testosterone concentrations in men, and thyroid hormone replacement has been found to normalise the testosterone concentrations (57). Furthermore, many hypothyroidism patients experience fatigue and fatigue-related symptoms, despite apparently adequate replacement therapy (58, 59). Since Physta^®^ 100 mg can increase T3 levels, this might contribute to an increase in the testosterone level and alleviate fatigue. However, there were no significant changes in thyroid hormones for Physta^®^ 200 mg supplementation, so the effect is not dose-dependent, possibly because of the adaptogenic effect of EL which establishes homeostasis according to the body’s needs. Further investigation is required to determine how EL modulates thyroid hormones.

Elevated cortisol levels, an indicator of high stress, is associated with lower testosterone concentrations (60). The within-group significant reduction observed in the Physta^®^ 200 mg group in serum cortisol levels and the non-significant decline observed in the Physta^®^ 100 mg group suggest that the effect of Physta^®^ on cortisol is dose-dependent. In a previous study, daily supplementation of Physta^®^ significantly lowered the cortisol levels in moderately stressed subjects compared to placebo (25). A between-group significant reduction in cortisol levels was seen in the previous study (25) but not in this study. This may be due to a difference in the study population, because moderately stressed subjects were involved in the previous study while this study did not involve stressed subjects.

Deteriorating muscle strength and function that occurs in ageing men is shown to be related to a decline in the serum testosterone (61). This study found that a significant within-group improvement in muscle tone in both treatment groups compared to baseline confirms the observation of a previous study that proved EL, particularly Physta^®^, to be ergogenic by enhancing muscle strength in elderly people supplemented with 400 mg Physta^®^ daily for 5 weeks (26).

In terms of safety, there were no significant and sustained changes compared to placebo in blood, liver and kidney laboratory tests. Significant changes were noted at the end of week 12 in some of the white blood cell components as eosinophil, lymphocytes, monocytes and basophil. The changes are not dose-dependent and are also seen in the placebo group and therefore could not derive a conclusion on if Physta^®^ has affected these parameters; it may be contributed by other factors as diet and lifestyle (62). All other significant changes were not dose-dependent and within acceptable clinical ranges. Some common gastrointestinal symptoms were noted in Physta^®^ group; according to case report files, the symptoms were mild and lasted for 3–5 days, and the subjects were recovered without any treatment. Gastrointestinal symptoms are commonly caused by other plant extracts as cinnamon (63), ginseng and guarana (64) too. Generally, the Physta^®^ was considered as safe and well tolerated in the study population, with no reported SAEs, in line with previous findings from randomised and controlled clinical studies evaluating Physta^®^ (25, 26, 31, 33, 37, 45). Vital signs and anthropometric measurements also showed no significant changes.

Daily food intake, exercise regime and other lifestyle details of the subjects were not collected in the study. Some of this could be confounding factors that may have an effect on the Qol parameters. In future studies, these data should be collected or controlled to minimize variables that can affect the study results. EL is known to be used traditionally in the treatment of gastrointestinal symptoms (65) but mild gastrointestinal symptoms were observed in Physta^®^ groups; this conflicting finding should be further investigated in future studies with bigger study population as this study has the limitation of involving a small number of subjects.

## Conclusion

The process of ageing and the deteriorating physiological functions are inevitable but they can be delayed or their ill effects reduced. This study demonstrated that Physta^®^ supplementation as low as 100 and 200 mg in aged male subjects with low testosterone levels resulted in significant improvements in the total testosterone levels, accompanied by enhanced QoL scores and reduced ageing symptoms and fatigue as early as 4 and 2 weeks of supplementation, respectively.

## Data Availability

The datasets used and/or analysed during this study are available from the corresponding author on reasonable request.
